# K_9_Y_3_[Si_12_O_32_]F_2_

**DOI:** 10.1107/S1600536814001470

**Published:** 2014-01-25

**Authors:** Volker Kahlenberg, Tanja Manninger

**Affiliations:** a University of Innsbruck, Institute of Mineralogy & Petrography, Innrain 52, A-6020 Innsbruck, Austria

## Abstract

Single-crystals of the title compound, nona­potassium triyttrium dodeca­silicate difluoride, were obtained from flux synthesis experiments in the system SiO_2_—Y_2_O_3_—KF. The crystal structure belongs to the group of single-layer silicates and is based on silicate sheets parallel to (110). A single layer contains secondary (*Q* ^2^) and tertiary (*Q* ^3^) silicate tetra­hedra in the ratio 1:2 and is build up from six-, eight- and twelve-membered rings. The linkage between neighboring layers is achieved by two crystallographically independent Y^3+^ cations, which are coordinated by six oxygen ligands in form of distorted octa­hedra. Charge compensation is accomplished by incorporation of additional F^−^ anions and K^+^ cations in the structural channels, forming anion-centred [F_2_K_7_] groups. Apart from one K^+^ and one Y^3+^ cation (each with site symmetry -1), the 30 crystallographically independent atoms reside on general positions.

## Related literature   

Oxosilicates which can serve as luminescent materials containing trivalent rare earth elements, monovalent alkali cations as well as fluorine anions have already been characterized (Jacobsen & Meyer, 1994 ▶; Tang *et al.*, 2008 ▶; Schäfer & Schleid, 2007 ▶, 2011 ▶). For structures isotypic to that of the title compound, see: Tang *et al.* (2008 ▶). For general aspects of the crystal chemistry of silicates, see: Liebau (1985 ▶). For the definition of distortion parameters, see: Robinson *et al.* (1971 ▶). For bond-valence analysis, see: Brown & Altermatt (1985 ▶). For the Inorganic Crystal Structure Database, see: ICSD (2013 ▶).

## Experimental   

### 

#### Crystal data  

K_9_Y_3_[Si_12_O_32_]F_2_
*M* *_r_* = 1505.71
Triclinic, 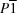

*a* = 6.8187 (3) Å
*b* = 11.3345 (4) Å
*c* = 11.3727 (5) Å
α = 87.846 (3)°
β = 89.747 (4)°
γ = 80.524 (3)°
*V* = 866.35 (6) Å^3^
*Z* = 1
Mo *K*α radiation
μ = 6.60 mm^−1^
*T* = 298 K
0.12 × 0.09 × 0.05 mm

#### Data collection  

Oxford Diffraction Xcalibur diffractometer
Absorption correction: multi-scan (*CrysAlis PRO*; Oxford Diffraction, 2006 ▶) *T*_min_ = 0.801, *T*_max_ = 1
10508 measured reflections
2842 independent reflections
2231 reflections with *I* > 2σ(*I*)
*R*_int_ = 0.066

#### Refinement  

*R*[*F* ^2^ > 2σ(*F* ^2^)] = 0.039
*wR*(*F* ^2^) = 0.099
*S* = 1.08
2842 reflections
265 parameters
Δρ_max_ = 0.87 e Å^−3^
Δρ_min_ = −0.95 e Å^−3^

### 

Data collection: *CrysAlis PRO* (Oxford Diffraction, 2006 ▶); cell refinement: *CrysAlis PRO*; data reduction: *CrysAlis PRO*; program(s) used to solve structure: *SIR2002* (Burla *et al.*, 2003 ▶); program(s) used to refine structure: *SHELXL97* (Sheldrick, 2008 ▶); molecular graphics: *ATOMS for Windows* (Dowty, 2011 ▶); software used to prepare material for publication: *publCIF* (Westrip, 2010 ▶) and *WinGX* (Farrugia, 2012 ▶).

## Supplementary Material

Crystal structure: contains datablock(s) global, I. DOI: 10.1107/S1600536814001470/wm2792sup1.cif

Structure factors: contains datablock(s) I. DOI: 10.1107/S1600536814001470/wm2792Isup2.hkl

Additional supporting information:  crystallographic information; 3D view; checkCIF report

## Figures and Tables

**Fig. 1.**
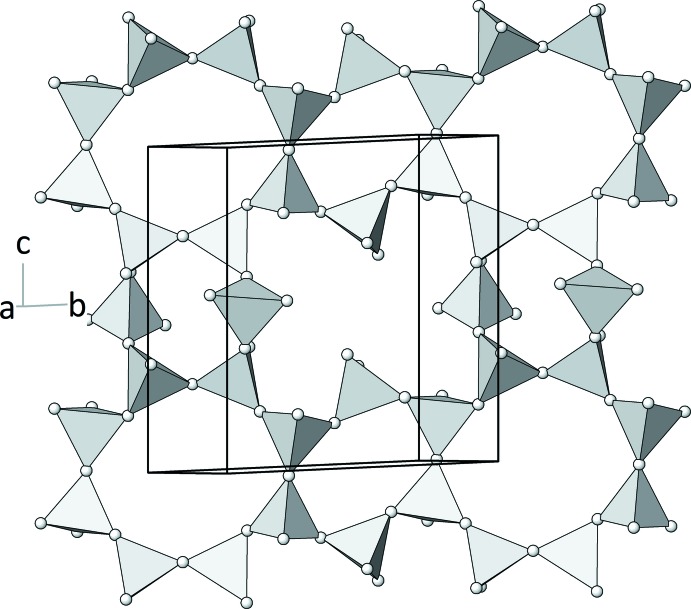
A single silicate layer consisting of [SiO4] tetrahedra in a projection perpendicular to (110).

**Fig. 2.**
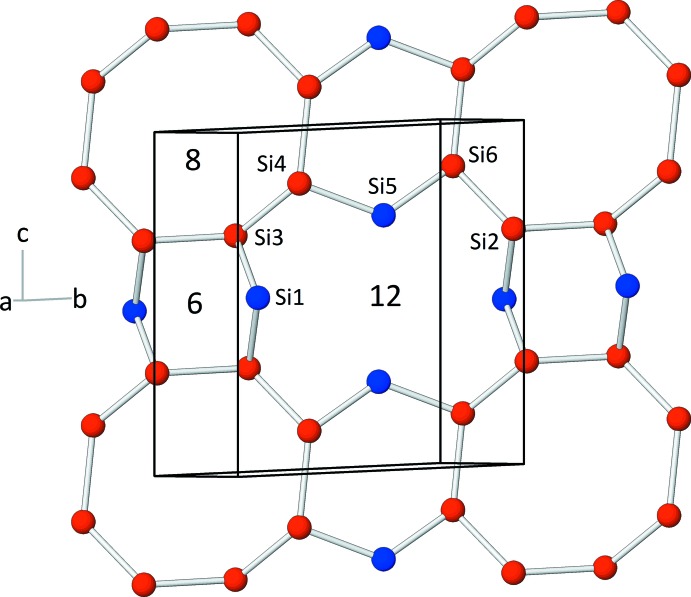
Connectivity of the silicon atoms within a single layer. Red and blue spheres represent Q3- and Q2-connected atoms, respectively. The sizes of the different ring types are indicated.

**Fig. 3.**
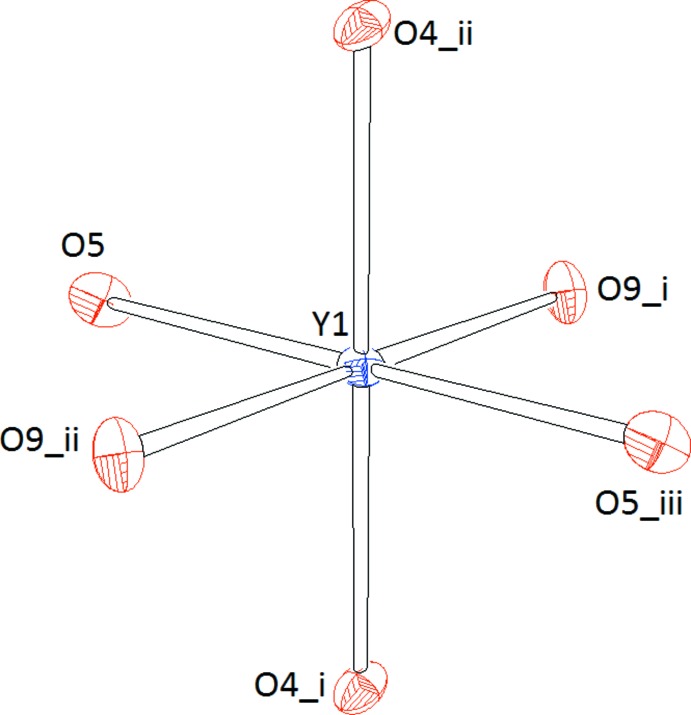
Representation of the coordination polyhedron around Y1. Ellipsoids are drawn at the 60% level. [Symmetry codes: (i) 1 - x,-y, 1 - z; (ii) -1 + x,y,z; (iii) -x,-y,1 - z; (iv) 1 - x,1 - y,1 - z; (v) x,1 + y,z; (vi) 1 + x,1 + y,z; (vii) 1 - x,-y,-z.]

**Fig. 4.**
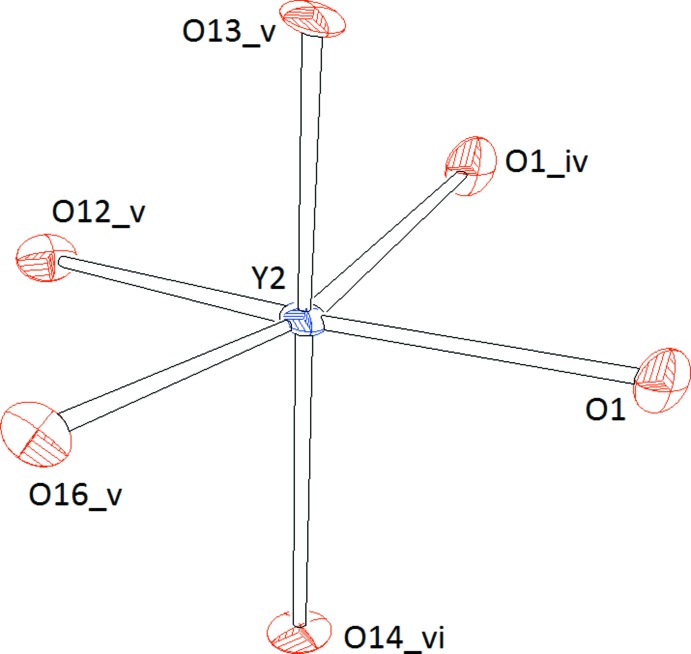
Representation of the coordination polyhedron around Y2. Ellipsoids are drawn at the 60% level. Symmetry codes: [(i) 1 - x,-y, 1 - z; (ii) -1 + x,y,z; (iii) -x,-y,1 - z; (iv) 1 - x,1 - y,1 - z; (v) x,1 + y,z; (vi) 1 + x,1 + y,z; (vii) 1 - x,-y,-z.]

**Fig. 5.**
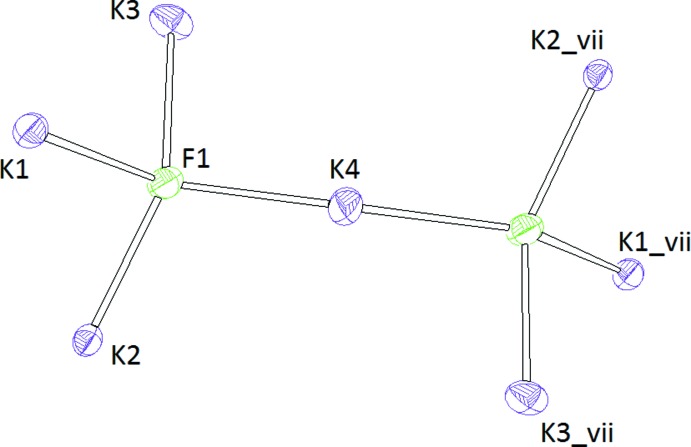
Representation of a single [F2K7]-group. Ellipsoids are drawn at the 60% level. [Symmetry codes: (i) 1 - x,-y, 1 - z; (ii) -1 + x,y,z; (iii) -x,-y,1 - z; (iv) 1 - x,1 - y,1 - z; (v) x,1 + y,z; (vi) 1 + x,1 + y,z; (vii) 1 - x,-y,-z.]

**Fig. 6.**
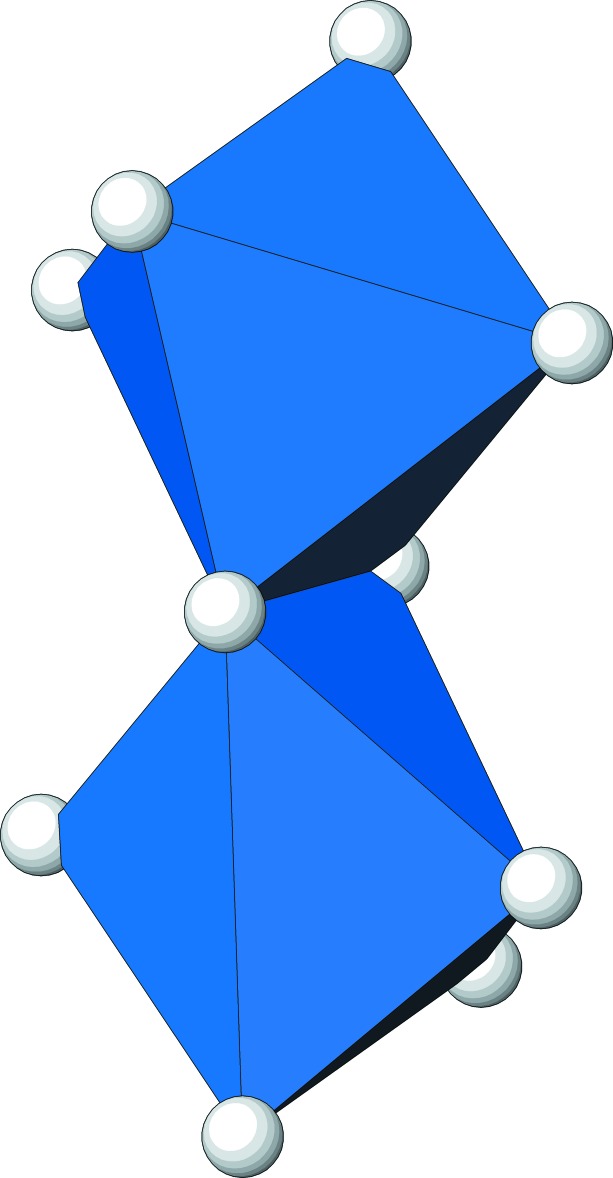
The dimer formed from the condensation of two edge-sharing [Y2O6]-octahedra.

**Fig. 7.**
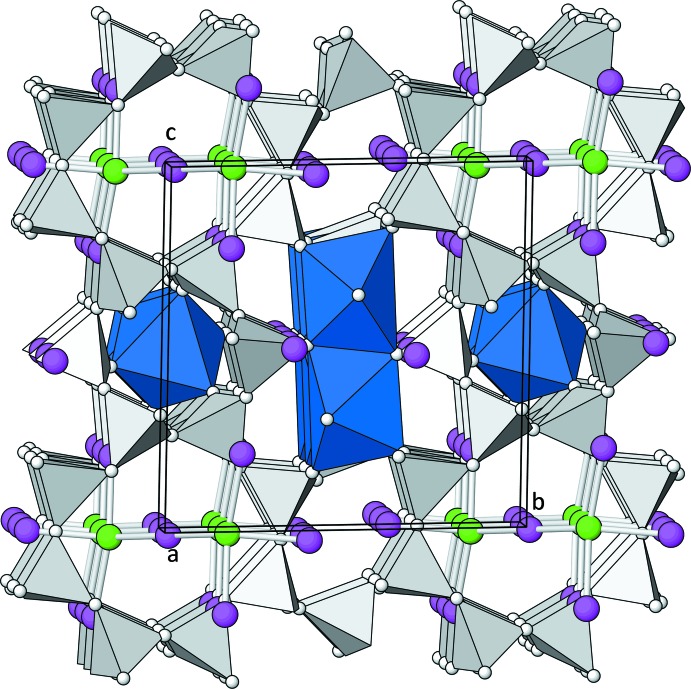
Side view of the whole crystal structure of K9Y3[Si12O32]F2. [SiO4]- and [YO6]-polyhedra are shown in light-grey and blue. Small grey spheres represent oxygen atoms. Fluorine and potassium ions are given as larger green and pink spheres. F—K bonds of the [F2K7]-double tetrahedra are indicated.

## supplementary crystallographic information

### 1. Comment

In the present paper we describe a previously unknown phase of the system KF—Y_2_O_3_—SiO_2_. According to Liebau's classification (1985) the crystal structure of the title compound, K_9_Y_3_[Si_12_O_32_]F_2_, belongs to the group of open-branched single-layer silicates. The more detailed crystallochemical formula can be written as K_9_Y_3_{*oB*,1^2^_∞_}[^5^Si_12_O_32_]F_2_. A single-layer of silicate tetrahedra expands parallel to (110) and is constructed from the condensation of *fünfer* single chains (Fig. 1). One discrete chain is running parallel to [001] and has a translation period of 11.3727 (5) Å. Each layer contains secondary (*Q*^2^) and tertiary (*Q*^3^) SiO_4_ tetrahedra in the ratio of 1:2 (Fig. 2). The Si—O distances of the six crystallographically independent tetrahedra within the asymmetric unit range from 1.574 (5) to 1.662 (5) Å. As one would expect, the Si—O_terminal_ bonds are considerably shorter than the distances between Si and the bridging O atoms. The O—Si—O angles show a significant scatter throughout all present polyhedra. Nevertheless, the values are in the expected limits for [SiO_4_] units (Liebau, 1985). Numerically, the degree of distortion can be expressed by the quadratic elongation λ and the angle variance σ^2^ (Robinson *et al.*, 1971). For the six tetrahedra, these two parameters vary between 1.003 and 1.005 (for λ) and 8.50 and 20.17 (for σ^2^) indicating that the deviation from regularity is not very pronounced.

Within the corrugated silicate sheets, six-, eight- and twelve-membered rings can be identified (Fig. 2). The vertex symbols for the [SiO_4_] tetrahedra are as follows: 6.8.12 (for Si2, Si3, Si4 and Si6) and 6.12 (for Si1 and Si5). A schematic representation of the arrangement of the rings within a single layer is given in Fig. 2. Charge balance in the structure is achieved by the incorporation of K^+^ and Y^3+^ cations as well as additional F^-^ anions. Y1 resides on an inversion center and is coordinated by six oxygen ligands belonging to six different [SiO_4_]-tetrahedra (Fig. 3). Within the resulting octahedron, the Y—O bond lengths range from 2.237 (4) - 2.256 (4) Å. Y2 is also octahedrally coordinated (Fig. 4). However, each two adjacent [Y2O_6_]-octahedra form dimers by sharing one common edge (Fig. 6). Therefore, the spread in the Y—O bond lengths is more siginificant (2.211 (4) - 2.345 (4) Å) which is also reflected in higher values for the distortion parameters: λ = 1.046 and σ^2^ = 149.49 (for Y2), and λ = 1.006 and σ^2^ = 20.88 (for Y1), respectively. The volumes of both octahedra are almost identical: 14.545 Å^3^ (for Y2) and 14.991 Å^3^ (for Y1). The coordination numbers of the potassium cations are as follows: K1, K2: 8-coordinate, including one F atom; K3: 7-coordinate, including one F atom; K4: 8-coordinate, including two F atoms; K5: 7-coordinate, only O atoms. A slightly different understanding of the structure can be obtained when anion-centred polyhedra are considered as well for the description. Actually, each F^-^ has four nearest potassium neighbors in form of a tetrahedron. Two symmetry-equivalent tetrahedra are joined by a common corner (K4) into [F_2_K_7_]-double tetrahedra with point group symmetry 1 (Fig. 5). A side view of the whole structure is given in Fig. 7.

Bond valence sum calculations using the parameter sets for the K—O, K—F, Y—O and Si—O bonds given by Brown & Altermatt (1985) resulted in the following values (in v.u.) considering cation—anion interactions up to 3.4 Å: K1: 0.924, K2: 0.957, K3: 0.844, K4: 0.772, K5: 1.057, Y1: 3.242, Y2: 3.087, Si1: 4.264, Si2: 4.257, Si3: 4.347, Si4: 4.342, Si5: 4.251, and Si6: 4.326.

The present compound is isostructural with a series of rare earth fluoride silicates: K_9_(*REE*)_3_[Si_12_O_32_]F_2_ (*REE*: Sm, Eu, Gd; Tang *et al.*, 2008). Chemically related compounds include the following phases: KEu_2_[Si_4_O_10_]F (Jacobsen & Meyer, 1994), Cs_2_Y[Si_4_O_10_]F (Schäfer & Schleid, 2007) and Rb_3_Sc_2_[Si_4_O_10_]F_5_ (Schäfer & Schleid, 2011).

### 2. Experimental

Single-crystals of K_9_Y_3_[Si_12_O_32_]F_2_ were obtained during a series of flux syntheses experiments aiming on the preparation of new K(*REE*)-silicate fluorides. 0.1 g of the nutrient consisting of a mixture of Y_2_O_3_:SiO_2_ in the molar ratio 1:4 was homogenized in an agate mortar with 0.1 g KF, transferred into a platinum tube and welded shut. The container was fired in a resistance heated furnace from 373 K to 1373 K with a ramp of 50 K/h. The target temperature was held for 2 h. Subsequently, the sample was cooled down to 1073 K with a rate of 5 K/h and, finally, the temperature was reduced to 373 K with a rate of 100 K/h. The solidified melt cake was immediately crushed in an agate mortar and transferred to a glass slide under a polarizing binocular. A first optical inspection revealed the presence of two phases: a polycrystalline matrix of KF in which transparent birefrigent single-crystals up to 200 µm in size were embedded. However, a closer investigation using crossed polarizers revealed that all crystals showed a fine-scale non-merohedral twinning, making it impossible to separate a specimen consisting of only one domain state. Therefore, we finally decided to use a twinned fragment for further structural studies. The crystal was mounted on the tip of a glass fibre using finger nail hardener as glue.

### 3. Refinement

The diffraction patterns were collected at ambient temperature using on Oxford Diffraction Gemini R Ultra single-crystal diffractometer. They showed the expected complexity due to overlapping of two different reciprocal lattices. Nevertheless, it was possible to index the reflections from both domains with the same triclinic unit cell but in different orientations. From the fact that the angle β is close to 90°, the non-merohedral twinning can be readily understood. Similar sets of lattice parameters could be found in the recent WEB-based version of the Inorganic Crystal Structure Database (ICSD, 2013) for the chemically closely related compounds K_9_(*REE*)_3_[Si_12_O_32_]F_2_ (*REE* = Sm, Eu, Gd) pointing to an isostructural relationship, which was confirmed by the subsequent structure analysis. For structure determination a full data set (sphere) of reciprocal space was collected. Different integration strategies were tested to handle the problem of the partially overlapping reflections of both domains, *i.e.* a series of data sets was produced in which the overlap threshold was varied stepwise. Different HKLF 5 data sets produced during integration were considered for the refinement of the structure. However, the best results concerning residuals and overall crystallochemical characteristics of the structure were obtained when the data set of only the main twin component (representating about 70% of the total volume) was used, *i.e.* the completely or partially overlapping reflections have been neglected. However, this approach resulted in a completeness of only 90%.

### Crystal data

**Table:**

F_2_K_9_O_32_Si_12_Y_3_	*Z* = 1
*M**_r_* = 1505.71	*F*(000) = 730
Triclinic, *P*1	*D*_x_ = 2.886 Mg m^−^^3^
Hall symbol: -P 1	Mo *K*α radiation, λ = 0.71073 Å
*a* = 6.8187 (3) Å	Cell parameters from 4373 reflections
*b* = 11.3345 (4) Å	θ = 3.0–29.3°
*c* = 11.3727 (5) Å	µ = 6.60 mm^−^^1^
α = 87.846 (3)°	*T* = 298 K
β = 89.747 (4)°	Fragment, colourless
γ = 80.524 (3)°	0.12 × 0.09 × 0.05 mm
*V* = 866.35 (6) Å^3^	

### Data collection

**Table:**

Oxford Diffraction Xcalibur diffractometer	2842 independent reflections
Graphite monochromator	2231 reflections with *I* > 2σ(*I*)
Detector resolution: 10.3575 pixels mm^-1^	*R*_int_ = 0.066
ω scans	θ_max_ = 25.4°, θ_min_ = 3.3°
Absorption correction: multi-scan (*CrysAlis PRO*; Oxford Diffraction, 2006)	*h* = −8→8
*T*_min_ = 0.801, *T*_max_ = 1	*k* = −13→13
10508 measured reflections	*l* = −13→13

### Refinement

**Table:**

Refinement on *F*^2^	0 restraints
Least-squares matrix: full	Primary atom site location: structure-invariant direct methods
*R*[*F*^2^ > 2σ(*F*^2^)] = 0.039	Secondary atom site location: difference Fourier map
*wR*(*F*^2^) = 0.099	*w* = 1/[σ^2^(*F*_o_^2^) + (0.0515*P*)^2^ + 0.2439*P*] where *P* = (*F*_o_^2^ + 2*F*_c_^2^)/3
*S* = 1.08	(Δ/σ)_max_ < 0.001
2842 reflections	Δρ_max_ = 0.87 e Å^−^^3^
265 parameters	Δρ_min_ = −0.95 e Å^−^^3^

### Special details

**Table:**

Geometry. All s.u.'s (except the s.u. in the dihedral angle between two l.s. planes) are estimated using the full covariance matrix. The cell s.u.'s are taken into account individually in the estimation of s.u.'s in distances, angles and torsion angles; correlations between s.u.'s in cell parameters are only used when they are defined by crystal symmetry. An approximate (isotropic) treatment of cell s.u.'s is used for estimating s.u.'s involving l.s. planes.
Refinement. Refinement of *F*^2^ against ALL reflections. The weighted *R*-factor *wR* and goodness of fit *S* are based on *F*^2^, conventional *R*-factors *R* are based on *F*, with *F* set to zero for negative *F*^2^. The threshold expression of *F*^2^ > 2σ(*F*^2^) is used only for calculating *R*-factors(gt) *etc*. and is not relevant to the choice of reflections for refinement. *R*-factors based on *F*^2^ are statistically about twice as large as those based on *F*, and *R*- factors based on ALL data will be even larger.

### Fractional atomic coordinates and isotropic or equivalent isotropic displacement parameters (Å^2^)

**Table:**

	*x*	*y*	*z*	*U*_iso_*/*U*_eq_	
K1	0.2651 (3)	−0.38667 (12)	0.01644 (15)	0.0222 (4)	
K2	0.1715 (2)	−0.19454 (12)	−0.22364 (14)	0.0184 (4)	
K3	0.1624 (3)	−0.15506 (14)	0.21410 (15)	0.0277 (4)	
K4	0.5	0	0	0.0334 (6)	
K5	0.0227 (2)	0.33078 (11)	0.51133 (13)	0.0161 (3)	
F1	0.2472 (7)	−0.1589 (3)	−0.0048 (4)	0.0256 (10)	
Y1	0	0	0.5	0.0068 (2)	
Y2	0.48856 (9)	0.48851 (5)	0.33734 (5)	0.00691 (17)	
Si1	0.5571 (3)	0.23257 (13)	0.51056 (15)	0.0072 (4)	
Si2	0.3376 (3)	0.13524 (14)	0.31036 (16)	0.0077 (4)	
Si3	0.6564 (3)	−0.09115 (13)	0.30291 (15)	0.0071 (4)	
Si4	0.6897 (3)	−0.30506 (13)	0.15893 (16)	0.0076 (4)	
Si5	−0.0138 (3)	−0.51249 (14)	0.25833 (15)	0.0077 (4)	
Si6	0.2872 (3)	−0.66727 (14)	0.12015 (16)	0.0079 (4)	
O1	0.5290 (7)	0.3756 (3)	0.5151 (4)	0.0115 (10)	
O2	0.4907 (6)	0.2083 (3)	0.3742 (4)	0.0113 (10)	
O3	0.3903 (6)	0.1857 (3)	0.5954 (4)	0.0097 (9)	
O4	0.7743 (6)	0.1627 (3)	0.5395 (4)	0.0107 (10)	
O5	0.1448 (7)	0.1240 (3)	0.3837 (4)	0.0125 (10)	
O6	0.2791 (7)	0.2030 (3)	0.1831 (4)	0.0129 (10)	
O7	0.4575 (7)	0.0054 (3)	0.2739 (4)	0.0132 (10)	
O8	0.6920 (7)	−0.1638 (3)	0.1818 (4)	0.0102 (10)	
O9	0.8444 (7)	−0.0336 (3)	0.3338 (4)	0.0114 (10)	
O10	0.9033 (7)	−0.3743 (3)	0.2102 (4)	0.0139 (10)	
O11	0.6983 (7)	−0.3156 (3)	0.0189 (4)	0.0169 (11)	
O12	0.5030 (7)	−0.3527 (3)	0.2148 (4)	0.0123 (10)	
O13	0.1635 (7)	−0.5084 (3)	0.3462 (4)	0.0114 (10)	
O14	−0.1887 (6)	−0.5722 (3)	0.3149 (4)	0.0121 (10)	
O15	0.0686 (7)	−0.5849 (3)	0.1393 (4)	0.0131 (10)	
O16	0.4610 (7)	−0.6045 (3)	0.1658 (4)	0.0123 (10)	

### Atomic displacement parameters (Å^2^)

**Table:**

	*U*^11^	*U*^22^	*U*^33^	*U*^12^	*U*^13^	*U*^23^
K1	0.0222 (9)	0.0215 (8)	0.0233 (10)	−0.0056 (6)	0.0004 (7)	0.0030 (6)
K2	0.0165 (9)	0.0192 (7)	0.0197 (9)	−0.0036 (6)	0.0005 (7)	0.0013 (6)
K3	0.0204 (10)	0.0421 (10)	0.0197 (9)	−0.0011 (7)	0.0057 (8)	−0.0068 (7)
K4	0.0395 (17)	0.0363 (13)	0.0280 (15)	−0.0195 (11)	−0.0105 (12)	0.0117 (10)
K5	0.0190 (9)	0.0132 (7)	0.0158 (9)	−0.0020 (6)	0.0035 (7)	−0.0012 (5)
F1	0.030 (3)	0.026 (2)	0.022 (2)	−0.0079 (18)	0.004 (2)	−0.0013 (17)
Y1	0.0038 (5)	0.0078 (4)	0.0086 (5)	−0.0007 (3)	0.0021 (4)	−0.0001 (3)
Y2	0.0032 (3)	0.0093 (3)	0.0082 (3)	−0.0010 (2)	0.0010 (2)	0.0001 (2)
Si1	0.0056 (10)	0.0072 (8)	0.0087 (10)	−0.0010 (6)	0.0009 (7)	0.0006 (6)
Si2	0.0053 (10)	0.0080 (8)	0.0099 (9)	−0.0017 (6)	0.0002 (7)	−0.0003 (6)
Si3	0.0038 (9)	0.0078 (8)	0.0101 (10)	−0.0015 (6)	0.0015 (7)	−0.0010 (6)
Si4	0.0064 (10)	0.0076 (8)	0.0089 (9)	−0.0012 (6)	0.0010 (7)	−0.0011 (6)
Si5	0.0035 (9)	0.0104 (8)	0.0087 (9)	0.0002 (6)	0.0009 (7)	−0.0005 (6)
Si6	0.0072 (10)	0.0084 (8)	0.0081 (10)	−0.0014 (6)	0.0014 (7)	−0.0006 (6)
O1	0.017 (3)	0.006 (2)	0.011 (3)	−0.0005 (17)	0.002 (2)	−0.0002 (16)
O2	0.008 (3)	0.014 (2)	0.014 (3)	−0.0057 (17)	−0.001 (2)	−0.0002 (17)
O3	0.009 (3)	0.012 (2)	0.008 (2)	−0.0029 (17)	0.0027 (19)	−0.0004 (17)
O4	0.005 (2)	0.016 (2)	0.011 (3)	−0.0002 (17)	0.0034 (19)	0.0001 (17)
O5	0.010 (3)	0.013 (2)	0.016 (3)	−0.0037 (17)	0.000 (2)	−0.0016 (17)
O6	0.018 (3)	0.012 (2)	0.010 (3)	−0.0063 (18)	0.001 (2)	0.0035 (17)
O7	0.008 (3)	0.009 (2)	0.021 (3)	0.0039 (17)	−0.001 (2)	−0.0031 (18)
O8	0.015 (3)	0.007 (2)	0.008 (2)	−0.0011 (17)	0.003 (2)	0.0002 (16)
O9	0.008 (3)	0.016 (2)	0.011 (3)	−0.0053 (18)	−0.0006 (19)	0.0005 (17)
O10	0.004 (2)	0.012 (2)	0.025 (3)	−0.0003 (17)	−0.003 (2)	0.0032 (18)
O11	0.022 (3)	0.014 (2)	0.015 (3)	−0.0024 (19)	0.004 (2)	−0.0052 (18)
O12	0.009 (3)	0.010 (2)	0.017 (3)	−0.0022 (17)	0.004 (2)	0.0013 (17)
O13	0.006 (2)	0.017 (2)	0.011 (3)	−0.0028 (17)	0.002 (2)	0.0005 (17)
O14	0.004 (2)	0.013 (2)	0.019 (3)	−0.0020 (17)	−0.001 (2)	0.0021 (18)
O15	0.007 (3)	0.018 (2)	0.013 (3)	0.0015 (18)	0.002 (2)	−0.0040 (18)
O16	0.006 (3)	0.017 (2)	0.015 (3)	−0.0054 (17)	0.003 (2)	−0.0045 (17)

### Geometric parameters (Å, º)

**Table:**

K1—F1	2.567 (4)	Si1—O3	1.631 (5)
K1—O16^i^	2.778 (5)	Si1—O2	1.662 (5)
K1—O12	2.858 (5)	Si2—O5	1.574 (5)
K1—O15^ii^	2.956 (5)	Si2—O2	1.625 (4)
K1—O16	3.064 (5)	Si2—O7	1.629 (4)
K1—O15	3.088 (5)	Si2—O6	1.633 (5)
K1—O11	3.188 (5)	Si3—O9	1.578 (4)
K1—O10^iii^	3.289 (5)	Si3—O3^vi^	1.613 (4)
K1—O11^i^	3.382 (4)	Si3—O7	1.623 (4)
K2—F1	2.604 (4)	Si3—O8	1.628 (4)
K2—O9^iv^	2.816 (4)	Si4—O12	1.584 (5)
K2—O5^v^	2.821 (5)	Si4—O11	1.602 (5)
K2—O2^iv^	2.851 (5)	Si4—O10	1.635 (5)
K2—O14^ii^	2.861 (4)	Si4—O8	1.635 (4)
K2—O6^v^	3.121 (5)	Si5—O13	1.579 (5)
K2—O16^i^	3.150 (5)	Si5—O14	1.588 (4)
K2—O15^ii^	3.317 (4)	Si5—O10^iii^	1.649 (4)
K3—F1	2.554 (5)	Si5—O15	1.657 (5)
K3—O9^iii^	2.754 (5)	Si6—O16	1.578 (4)
K3—O4^vi^	2.833 (5)	Si6—O11^i^	1.601 (5)
K3—O12	2.949 (5)	Si6—O6^xi^	1.620 (4)
K3—O7	3.021 (4)	Si6—O15	1.642 (5)
K3—O8^iii^	3.248 (5)	O1—Y2^x^	2.310 (4)
K3—O10^iii^	3.279 (4)	O1—K5^xii^	3.321 (5)
K4—F1^iv^	2.692 (4)	O2—K2^iv^	2.851 (5)
K4—F1	2.692 (4)	O3—Si3^vi^	1.613 (4)
K4—O8^iv^	2.893 (4)	O4—Y1^xii^	2.256 (4)
K4—O8	2.893 (4)	O4—K5^xii^	2.759 (4)
K4—O7	3.129 (5)	O4—K3^vi^	2.833 (5)
K4—O7^iv^	3.129 (5)	O5—K2^v^	2.821 (5)
K4—O6^iv^	3.330 (4)	O6—Si6^vii^	1.620 (4)
K4—O6	3.330 (4)	O6—K2^v^	3.121 (5)
K5—O14^vii^	2.756 (5)	O8—K3^xii^	3.248 (5)
K5—O4^iii^	2.759 (4)	O9—Y1^xii^	2.247 (4)
K5—O13^viii^	2.771 (4)	O9—K3^xii^	2.754 (5)
K5—O5	2.810 (4)	O9—K2^iv^	2.816 (4)
K5—O13^vii^	2.842 (5)	O10—Si5^xii^	1.649 (4)
K5—O3	2.907 (5)	O10—K5^vi^	3.277 (5)
K5—O10^vi^	3.277 (5)	O10—K3^xii^	3.279 (4)
K5—O1^iii^	3.321 (5)	O10—K1^xii^	3.289 (5)
Y1—O5	2.237 (4)	O11—Si6^i^	1.601 (5)
Y1—O5^viii^	2.237 (4)	O11—K1^i^	3.382 (4)
Y1—O9^vi^	2.247 (4)	O12—Y2^xi^	2.250 (4)
Y1—O9^iii^	2.247 (4)	O13—Y2^xi^	2.213 (4)
Y1—O4^vi^	2.256 (4)	O13—K5^viii^	2.771 (4)
Y1—O4^iii^	2.256 (4)	O13—K5^xi^	2.842 (5)
Y2—O14^ix^	2.211 (4)	O14—Y2^xiii^	2.211 (4)
Y2—O13^vii^	2.213 (4)	O14—K5^xi^	2.756 (5)
Y2—O12^vii^	2.250 (4)	O14—K2^ii^	2.861 (4)
Y2—O16^vii^	2.275 (4)	O15—K1^ii^	2.956 (5)
Y2—O1^x^	2.310 (4)	O15—K2^ii^	3.317 (4)
Y2—O1	2.345 (4)	O16—Y2^xi^	2.275 (4)
Si1—O4	1.590 (5)	O16—K1^i^	2.778 (5)
Si1—O1	1.603 (4)	O16—K2^i^	3.150 (5)
			
O5—Y1—O5^viii^	180	O5—Si2—O7	112.2 (2)
O5—Y1—O9^vi^	96.02 (15)	O2—Si2—O7	109.0 (2)
O5^viii^—Y1—O9^vi^	83.98 (15)	O5—Si2—O6	110.5 (3)
O5—Y1—O9^iii^	83.98 (15)	O2—Si2—O6	107.2 (2)
O5^viii^—Y1—O9^iii^	96.02 (15)	O7—Si2—O6	102.9 (2)
O9^vi^—Y1—O9^iii^	180	O9—Si3—O3^vi^	111.7 (2)
O5—Y1—O4^vi^	94.15 (15)	O9—Si3—O7	114.3 (2)
O5^viii^—Y1—O4^vi^	85.85 (15)	O3^vi^—Si3—O7	109.8 (2)
O9^vi^—Y1—O4^vi^	93.05 (15)	O9—Si3—O8	110.7 (2)
O9^iii^—Y1—O4^vi^	86.95 (15)	O3^vi^—Si3—O8	106.9 (2)
O5—Y1—O4^iii^	85.85 (15)	O7—Si3—O8	102.9 (2)
O5^viii^—Y1—O4^iii^	94.15 (15)	O12—Si4—O11	112.3 (3)
O9^vi^—Y1—O4^iii^	86.95 (15)	O12—Si4—O10	114.1 (2)
O9^iii^—Y1—O4^iii^	93.05 (15)	O11—Si4—O10	106.7 (3)
O4^vi^—Y1—O4^iii^	180.0000 (10)	O12—Si4—O8	113.3 (2)
O14^ix^—Y2—O13^vii^	162.32 (14)	O11—Si4—O8	105.2 (2)
O14^ix^—Y2—O12^vii^	90.23 (16)	O10—Si4—O8	104.5 (2)
O13^vii^—Y2—O12^vii^	100.81 (16)	O13—Si5—O14	113.5 (2)
O14^ix^—Y2—O16^vii^	84.41 (16)	O13—Si5—O10^iii^	107.8 (2)
O13^vii^—Y2—O16^vii^	83.37 (16)	O14—Si5—O10^iii^	110.8 (2)
O12^vii^—Y2—O16^vii^	82.65 (15)	O13—Si5—O15	110.3 (2)
O14^ix^—Y2—O1^x^	103.85 (16)	O14—Si5—O15	109.2 (2)
O13^vii^—Y2—O1^x^	90.94 (16)	O10^iii^—Si5—O15	104.9 (2)
O12^vii^—Y2—O1^x^	85.09 (15)	O16—Si6—O11^i^	111.4 (3)
O16^vii^—Y2—O1^x^	165.26 (15)	O16—Si6—O6^xi^	114.0 (2)
O14^ix^—Y2—O1	84.94 (16)	O11^i^—Si6—O6^xi^	107.7 (2)
O13^vii^—Y2—O1	90.07 (16)	O16—Si6—O15	111.7 (2)
O12^vii^—Y2—O1	156.35 (15)	O11^i^—Si6—O15	104.5 (2)
O16^vii^—Y2—O1	119.73 (14)	O6^xi^—Si6—O15	106.9 (2)
O1^x^—Y2—O1	73.70 (14)	Si2—O2—Si1	137.7 (3)
O4—Si1—O1	115.9 (2)	Si3^vi^—O3—Si1	147.0 (3)
O4—Si1—O3	111.7 (2)	Si6^vii^—O6—Si2	137.6 (3)
O1—Si1—O3	108.3 (2)	Si3—O7—Si2	142.8 (3)
O4—Si1—O2	110.8 (2)	Si3—O8—Si4	129.8 (3)
O1—Si1—O2	104.0 (2)	Si4—O10—Si5^xii^	134.1 (3)
O3—Si1—O2	105.4 (2)	Si4—O11—Si6^i^	177.1 (3)
O5—Si2—O2	114.3 (2)	Si6—O15—Si5	127.5 (3)

Symmetry codes: (i) −*x*+1, −*y*−1, −*z*; (ii) −*x*, −*y*−1, −*z*; (iii) *x*−1, *y*, *z*; (iv) −*x*+1, −*y*, −*z*; (v) −*x*, −*y*, −*z*; (vi) −*x*+1, −*y*, −*z*+1; (vii) *x*, *y*+1, *z*; (viii) −*x*, −*y*, −*z*+1; (ix) *x*+1, *y*+1, *z*; (x) −*x*+1, −*y*+1, −*z*+1; (xi) *x*, *y*−1, *z*; (xii) *x*+1, *y*, *z*; (xiii) *x*−1, *y*−1, *z*.
